# Ginsenoside Rb1 mitigates radiation-induced intestinal injury through Treg expansion and IL-10–mediated epithelial regeneration

**DOI:** 10.1016/j.jgr.2026.101084

**Published:** 2026-06-19

**Authors:** Jeong-Won Kim, Jin-Hwa Kim, Seohee Jo, Han Byul Kim, Seung Bum Lee, Ki-Chun Yoo, Sang-Pil Choi, Seokjin Lee, Ji-Soo Jeong, Yeonghoon Son, Hae-June Lee, Hyosun Jang

**Affiliations:** a Division of Radiation Biomedical Research, Korea Institute of Radiological and Medical Science, 75 Nowon-ro, Nowon-gu, Seoul, 01812, Republic of Korea; b College of Veterinary Medicine (BK21 FOUR Program), Chungnam National University, 99 Daehak-ro, Daejeon, 34131, Republic of Korea; c Laboratory of Radiation Exposure & Therapeutics, National Radiation Emergency Medical Center, Korea Institute of Radiological and Medical Sciences, 75 Nowon-ro, Nowon-gu, Seoul, 01812, Republic of Korea; d College of Veterinary Medicine, Jeju National University, Jeju, Republic of Korea

**Keywords:** Ginsenoside Rb1, Radiation, Enteropathy, Regulatory T cell, IL-10, Wnt/β-catenin

## Abstract

**Background:**

Radiation-induced enteropathy represents a key limitation to recovery after accidental radiation exposure or radiotherapy, with few effective pharmacological treatments. We investigated the therapeutic potential of ginsenoside Rb1, a major saponin from *Panax ginseng*, in attenuating the radiation-induced intestinal injury.

**Methods:**

C57BL/6 mice were exposed to abdominal irradiation at a dose of 13.5 Gy and treated with Rb1. The epithelial integrity, bacterial translocation, and immune modulation were assessed. *In vitro*, rat intestinal epithelial cell line, IEC-6 were examined for proliferation and Wnt/β-catenin activation in response to Rb1 and IL-10. The role of IL-10 was validated using neutralization experiments.

**Results:**

Rb1 significantly alleviated radiation-induced crypt damage, villus shortening, bacterial translocation, and inflammation. It enhanced regulatory T (Treg) cell population and IL-10 secretion, which in turn activated Wnt/β-catenin signaling and promoted epithelial regeneration. *In vitro*, IL-10, but not Rb1 directly, promoted the proliferation of irradiated IEC-6 cells, whereas IL-10 neutralization abolished the mitigative effect of Rb1 in vivo.

**Conclusion:**

Rb1 mitigated radiation-induced enteropathy by upregulating Treg abundance and IL-10 signaling, which in turn activated epithelial regeneration with Wnt/β-catenin pathway activation, supporting its potential as a therapeutic agent for radiation-induced intestinal injury.

## Graphical abstract

**Figure:**
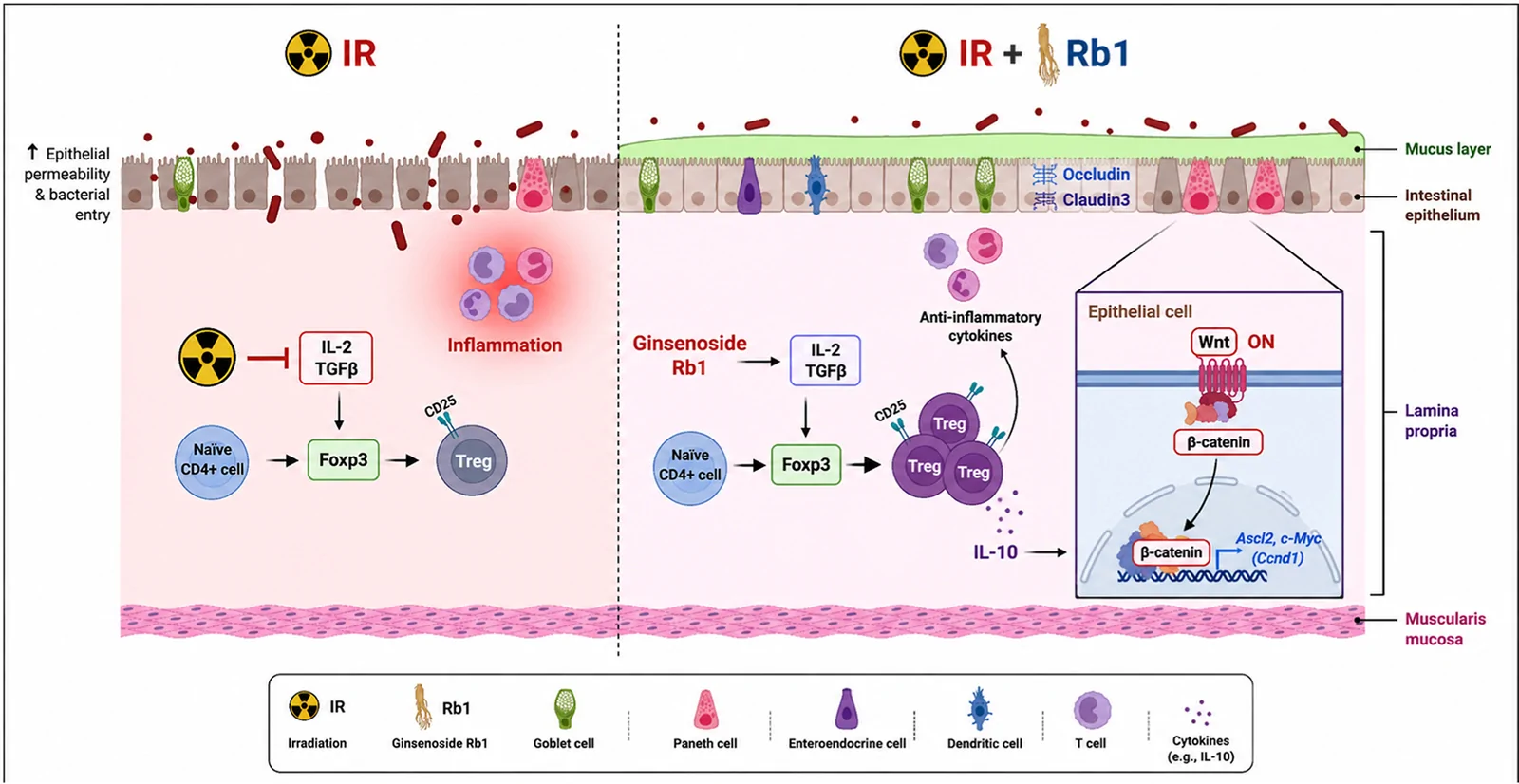

## 1 Introduction

Radiological accidents represent a persistent public health threat, with the capacity to induce mass casualty exposures and multi-organ injuries despite advances in radiation protection and safety [1]. Among organ systems, the intestinal epithelium is particularly vulnerable to irradiation due to its high radiosensitivity [2]. Consequently, radiation therapy inevitably injures adjacent normal intestinal tissues, thereby limiting therapeutic efficacy and causing acute and chronic toxicities that substantially diminishes patients’ quality of life [3]. Indeed, in murine models, exposure to 1 Gy of radiation can markedly elevate apoptotic cell death in the small intestine, occurring within 3–6 h after irradiation [4]. Radiation exposure to the intestine triggers multiple pathological events, including extensive epithelial cell death, loss of barrier integrity, villus atrophy, and inflammatory reactions [4]. This cascade of pathological events contributes to severe enterotoxicity, which clinically presents as gastrointestinal dysfunction, including nausea, diarrhea, bleeding, infection, and sepsis [5]. Despite these adverse effects, there are no FDA-approved pharmacological agents targeting radiation-induced gastrointestinal injury [6]. Therefore, strategies to prevent radiation-induced intestinal damage are urgently needed.

The intestinal epithelial layer performs multiple vital functions, including forming a protective barrier, enabling efficient nutrient uptake, interacting with resident microbiota, and orchestrating immune activity within the gut [7]. However, radiation exposure causes epithelial injury primarily through the depletion of intestinal stem cells (ISCs), suppression of epithelial proliferation, and disruption of tight junction integrity, collectively leading to compromised mucosal barrier function [8]. Epithelial tight junctions, composed of a complex network of transmembrane and cytoplasmic proteins, are essential for the barrier function of epithelial cells [9]. When radiation disrupts tight junctions or causes epithelial damage, the mucosa becomes abnormally permeable, a characteristic feature that contributes substantially to the development of radiation-associated enteropathy [10]. Therefore, promoting epithelial regeneration or strengthening the barrier function could serve as a therapeutic target for radiation-induced enteropathy [11,12].

Ginsenoside Rb1, one of the major triterpenoid saponins derived from *Panax ginseng*, is known to exert diverse biological activities, such as dampening inflammatory responses, counteracting oxidative stress, and protecting against neuronal injury [13,14]. Notably, Rb1 has been reported to suppress immune responses through the regulation of regulatory T (Treg) cells, and its therapeutic efficacy has been well demonstrated in various autoimmune diseases [15,16]. Additionally, Rb1 has been reported to alleviate ulcerative colitis in mice and rats by regulating inflammatory responses and epithelial integrity [13,17,18]. Previous studies have indicated that Rb1 supports intestinal stem cell activity and promotes epithelial expansion when the tissue is challenged by environmental or physiological stressors [19,20]. However, the cellular and molecular pathways by which Rb1 confers protection against radiation-induced intestinal injury remains unclear.

Based on these properties, we hypothesized that ginsenoside Rb1 could improve the epithelial regenerative properties against radiation-induced damage by modulating immune responses. In the present study, we investigated the therapeutic effects and underlying mechanisms of action of ginsenoside Rb1 in a mouse model of radiation-induced intestinal injury.

## 2 Materials and methods

### 2.1 Animals and ethics statement

Specific pathogen-free male C57BL/6 mice (6–7 weeks old) were obtained from Orient Bio (Seoul, Republic of Korea) and acclimated for 1 week. Mice were housed in the animal facility of Korea Institute of Radiological & Medical Sciences under controlled conditions (23 ± 1 °C; 12 h light/12 h dark cycle) with free access to food and water. The experimental groups were composed as follows: normal control (Con), irradiation only (IR), irradiation with Rb1 treatment (IR + Rb1; Cat. No. 15319, Cayman Chemical Company, Ann Arbor, MI, USA), and irradiation with Rb1 and anti-mouse IL-10 IgG antibody treatment (IR + Rb1 + anti-IL-10; clone JES5-2A5, Cat. No. BE0049, BioXCell, West Lebanon, NH, USA). All experimental procedures were approved by the Institutional Animal Care and Use Committee of the Korea Institute of Radiological & Medical Sciences (approval no. Kirams 2019-0051; Kirams2025-0013).

### 2.2 Irradiation and treatment

The mice were anesthetized via intraperitoneal injection of alfaxalone (85 mg/kg; Alfaxan, Careside, Gyeonggi-do, Republic of Korea) and xylazine (10 mg/kg; Rompun, Bayer Korea, Seoul, Republic of Korea). After anesthesia, abdominal irradiation was performed with a single dose of 13.5 Gy at a rate of 2 Gy/min using an X-RAD 320 X-ray irradiator (Softex, Gyeonggi-do, Republic of Korea). Rb1 (20 mg/kg) [21] or anti-mouse IL-10 antibody (5 mg/kg) was administered intraperitoneally once daily for up to 5 consecutive days after irradiation, with the first dose administered within 3 h after irradiation. In total, six doses were administered, and samples were collected 24 h after the final treatment.

### 2.3 Cell culture and proliferation assay

Rat intestinal epithelial IEC-6 cells (Cat. No. CRL-1592, ATCC, Manassas, VA, USA) were cultured with Dulbecco's modified eagle medium in 5% CO_2_ incubator (Sanyo, Tokyo, Japan). The cells were irradiated with 2 Gy or 4 Gy using a^137^Cs γ-ray source (Atomic Energy of Canada, Chalk River, ON, Canada) and subsequently treated with Rb1 or rat recombinant IL-10 (rIL-10; Cat. No. 400-19, PeproTech, NJ, USA) for 72 h (*n* = 3). Cell viability was evaluated using an EZ-Cytox Cell Proliferation Assay Kit (Cat. No. EZ-1000, DoGenBio, Seoul, Republic of Korea).

### 2.4 Colony forming assay

IEC-6 cells were seeded in six-well plates in triplicate at a density of 300 cells per well. The cells were exposed to 0, 2, or 4 Gy X-ray radiation and treated with or without 100 μΜ Rb1 or 100 ng/mL rIL-10. Cultures were maintained at 37 °C for 7 days to allow colony formation. Colonies were fixed with 4% paraformaldehyde for 30 min, stained with 0.04% crystal violet, and visualized under a microscope. Viable colonies containing more than 50 cells were counted.

### 2.5 Bacterial translocation and FITC–dextran permeability assays

On day 6 after irradiation, mesenteric lymph nodes (MLNs) were harvested, homogenized in sterile PBS, and centrifuged at 3000 × g for 5 min. The supernatant was plated on MacConkey agar (Cat. No. 211387, BD Biosciences, San Jose, CA, USA), incubated for 24 h, and colony formation was assessed as bacterial translocation. Because MacConkey agar selectively detects Gram-negative enterobacteria, this assay reflects a subset of translocated bacteria rather than the total bacterial burden. For intestinal permeability, mice were anesthetized and subjected to laparotomy. A 5-cm distal ileal segment was occluded with a bulldog clamp, and 100 μL of FITC–dextran (125 mg/mL, 4 kDa; Cat. No. 68059, Sigma-Aldrich, St. Louis, MO, USA) was injected intraluminally. After 30 min, blood was collected by cardiac puncture, and serum fluorescence was measured using a microplate reader (Thermo Fisher Scientific, Cleveland, OH, USA; excitation 485 nm, emission 528 nm).

### 2.6 Flow cytometry

Cells from the small intestinal lamina propria were isolated using Lamina Propria Dissociation Kit (Cat. No. 130-097-410, Miltenyi Biotec, Bergisch Gladbach, Germany) according to manufacturer's instructions and lymphocytes were purified using 40/80% Percoll concentration gradient. The cells were washed twice with Staining Buffer (Cat. No. 554656, BD Biosciences) and analyzed using a BD Accuri™ C6 Plus Flow Cytometer (BD Biosciences). Treg cells were identified as CD4, CD25, forkhead box P3 (FOXP3) positive. The fluorescent-conjugated antibodies were used as follows: Fluorescein isothiocyanate (FITC) anti-mouse CD4 IgG (Cat. No. 116003, BioLegend, San Diego, CA, USA), APC anti-mouse CD25 IgG (Cat. No. 102011, BioLegend), and PE anti-mouse FOXP3 (Cat. No. 126403, BioLegend).

### 2.7 Histological and immunofluorescence analysis of the intestine

Formalin-fixed small intestine tissues were embedded in paraffin and sectioned at 4 μm. Sections were deparaffinized, rehydrated, and stained with hematoxylin and eosin (H&E) according to standard protocols. Four fields per mouse from six mice per group were evaluated to measure villus length (n = 15 villi per sample) and crypt number per circumference. Radiation-induced enteritis severity was assessed based on inflammatory cell infiltration, vascular enlargement, crypt loss, and preservation of epithelial architecture. Immunohistochemistry (IHC) was performed as previously described [11]. After antigen retrieval with 0.3% hydrogen peroxide for 20 min and blocking with 10% normal goat serum (Cat. No. S-1000-20, Vector Laboratories, Nottingham, England, UK), sections were incubated with primary antibodies against villin (Cat. No. ab97512, Abcam, Cambridge, MA, USA), claudin-3 (Cat. No. 34-1700, Invitrogen, Carlsbad, CA, USA), Ki-67 (Cat. No. ab16667, Abcam), OLFM4 (Cat. No. 39141, Cell Signaling Technology, Danvers, MA, USA), active β-catenin (Cat. No. 19807, Cell Signaling Technology), CD68 (Cat. No. ab283654, Abcam), and MPO (Cat. No. ab208670, Abcam). Slides were then treated with horseradish peroxidase–conjugated secondary antibodies (Cat. No. P0448, DAKO, Hamburg, Germany) and developed with DAB, followed by hematoxylin counterstaining. For immunofluorescence (IF), deparaffinized sections were permeabilized with PBS containing 0.1% Triton X-100, blocked for 30 min, and incubated with goat anti-FOXP3 (Cat. No. NB100-39002, Novus Biologicals, Littleton, CO, USA) and rat anti–IL-10 (Cat. No. ab9969, Abcam) antibodies (1:200). Alexa Fluor 488–conjugated anti-rat IgG and Alexa Fluor 632–conjugated anti-goat IgG (Cat. No. A-11006, A-11055, Thermo Fisher Scientific) were applied for 1 h, and slides were mounted with DAPI (Cat. No. ab104139, Abcam). Random fields were examined under a light microscope at 100× and 200× magnifications.

### 2.8 Western blot

Cells were lysed in radioimmunoprecipitation assay buffer containing protease and phosphatase inhibitors (Cat. No. S8850, Sigma-Aldrich). Lysates were centrifuged at 12,000 × g for 10 min, and the supernatants were collected. Proteins were separated by SDS–PAGE and transferred to nitrocellulose membranes (Cat. No. 10600001, GE Healthcare, Piscataway, NJ, USA). After blocking, membranes were incubated with primary antibodies (1:1000) against active β-catenin, total β-catenin, axis inhibition protein 2 (AXIN2), and GAPDH (Cat. No. 2118, Cell Signaling Technology), and CCND1 (Cat. No. sc-8396, Santa Cruz Biotechnology, Santa Cruz, CA, USA). Protein bands were visualized using an Amersham ChemiDoc System (Amersham Lifesciences, Amersham, UK) and quantified using ImageJ software (NIH, Bethesda, MD, USA).

### 2.9 ELISA

Ileal tissues were homogenized in ice-cold lysis buffer and centrifuged to obtain tissue supernatants. The concentration of IL-10 in ileal tissue homogenates was measured using a mouse IL-10 DuoSet ELISA Development kit (Cat. No. DY417, R&D Systems, Minneapolis, MN, USA) according to the manufacturer's protocol. Briefly, microplates were coated with capture antibody overnight at room temperature, blocked with 1% BSA in PBS for 1 h, and incubated with standards and samples for 2 h at room temperature. After washing, detection antibody and streptavidin-HRP were applied sequentially, followed by TMB substrate development. The reaction was stopped with stop solution, and optical density was measured at 450 nm with correction at 540 nm. IL-10 concentrations were calculated from a 7-point, 2-fold serially diluted standard curve and normalized to total protein content.

### 2.10 Quantitative polymerase chain reaction (qPCR)

Total RNA was extracted from cell lysis using RNeasy® Total RNA Kit (Qiagen, Chatsworth, CA, USA) and transcribed using cDNA synthesis kit from Bioneer (Daejeon, Republic of Korea). qPCR was performed utilizing a SYBR Green PCR kit from Meridian Bioscience (Cincinnati, OH, USA) and a LightCycler 480 system (Roche, Basel, Switzerland). Primer sequences used for qPCR were as follows: For mouse, the primers targeted Cldn3 (forward AAGCCGAATGGACAAAGAA; reverse CTGGCAAGTAGCTGCAGTG), Villin (CACCTTTGGAAGCTTCTTCG; CTCTCGTTGCCTTGAACCTC), Ocln (TGGCAAGCGATCATACCCAGAG; CTGCCTGAAGTCATCCACACTC), Myc (TCGCTGCTGTCCTCCGAGTCC; GGTTTGCCTCTTCTCCACAGAC), Sox9 (CTCGCATACCTCCCTTCC; TTCCAGCAGTCACTAGGC), Ascl2 (TTTCCTGTGCCGCACCAGAACT; CAGCGACTCCAGACGAGGTGG), Ccnd1 (GCAGAAGGAGATTGTGCCATCC; AGGAAGCGGTCCAGGTAGTTCA), Lgr5 (AGAGCCTGATACCATCTGCAAAC; TGAAGGTCGTCCACACTGTTGC), Olfm4 (GCCTCCAAAAGTGACCTTGTGC; TGCGTGTGCTGGTGGAAAAGAG), Il1β (GCAACTGTTCCTGAACTCA; CTCGGAGCCTGTAGTGCAG), Il6 (AGCCAGAGTCCTTCAGAGAG; GATGGTCTTGGTCCTTAGCC), Tnfα (GCCTCTTCTCATTCCTGCTT; CACTTGGTGGTTTGCTACGA), Tbx21 (CCACCTGTTGTGGTCCAAGTTC; CCACAAACATCCTGTAATGGCTTG), Gata3 (CCTCTGGAGGAGGAACGCTAAT; GTTTCGGGTCTGGATGCCTTCT), Rorc (GCGACTGGAGGACCTT CTAC; TCCCACATTGACTTCCTCTG), Il2 (GCGGCATGTTCTGGATTTGACTC; CCACCACAGTTGCTGACTCATC), Tgfβ (CACTGATACGCCTGAGTG; CATCCCGTGGCTTCTAGTGC), Foxp3 (CCTGGTTGTGAGAAGGTCTTCG; TGCTCCAGAGACTGCACCACTT), Il10 (CGGGAAGACAATAACTGCACCC; CGGTTAGCAGTATGTTGTCCAGC), and Fgf2 (AAGCGGCTCTACTGCAAGAACG; CCTTGATAGACACAACTCCTCTC). Mouse Gapdh primers were AAGATGGTGATGGGCTTCCCG (forward) and TGGCAAAGTGGAGATTGTTGCC (reverse).

For rat, primers included Myc (GTGCTGCATGAAGAGACACC; CAACCTCTGGCTTCCTACCC), Sox9 (GCAAACACGTTGCAAATGGC; AACTCTGAAGGAGCCAAGCC), Ascl2 (TCTCTGTC CTGCACCTCTACATCC; AGCTGCTGTCCTCCGACGAG), Ccnd1 (ATGCTAGAGGTCTGCGAGGA; GGCTCCAGAGACAAGAAACG), and Gapdh (GAAGGTGAAGGTCGGAGTC; GAAGATGGTGATGGGATTTC).

### 2.11 Statistical analysis

Statistical significance between groups was determined using the GraphPad InStat software (version 8.0; GraphPad Software, Inc., La Jolla, CA, USA). The data are expressed as mean ± standard deviation, and statistical analyses were performed using one-way ANOVA followed by Tukey's post hoc test. Significance was defined by *^,#^*p* < 0.05.

## 3 Results

### 3.1 Ginsenoside Rb1 treatment attenuated acute radiation-induced intestinal injury with improved barrier function in a mouse model

**Fig. 1:**
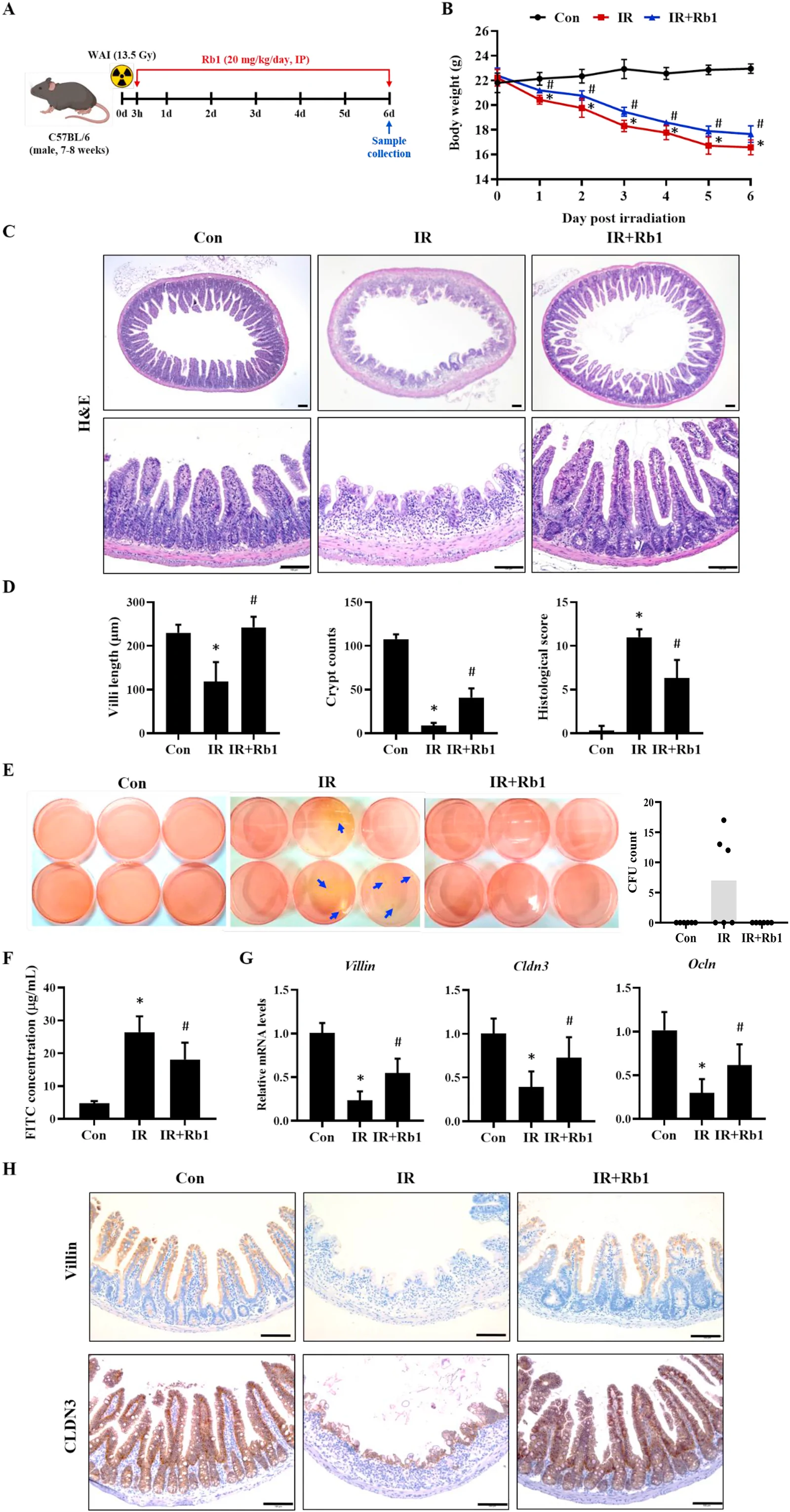
Ginsenoside Rb1 treatment attenuated acute radiation-induced intestinal injury with improved barrier function in a mouse model. (A) Experimental scheme. C57BL/6 mice received 13.5 Gy abdominal irradiation followed by daily Rb1 (20 mg/kg, i.p.) for 6 days. Tissues were collected 24 h after the final dose. (B) Body weight changes of control (Con), irradiated (IR), and Rb1-treated irradiated mice (IR + Rb1). (C) H&E staining of the small intestine. Scale bar = 100 μm. (D) Villi length, crypt counts, and histological score of the small intestine. (E) Representative images of bacterial colonies cultured from mesenteric lymph nodes and quantification of colony-forming units (CFUs) per mesenteric lymph nodes sample. (F) Plasma concentration of FITC derived from leakage across the intestinal lumen. (G) mRNA levels of *Villin, Cldn3,* and *Ocln* in the small intestine. (H) Representative IHC staining of villin and CLDN3 in the small intestine. Scale bar = 100 μm. Values are shown as means ± standard deviations (*n* = 6). Statistical significance was determined using one-way ANOVA followed by Tukey's post hoc test. *p < 0.05 vs. Con group; ^#^*p* < 0.05 vs. IR group.

To investigate the therapeutic effects of Rb1 on radiation-induced enteropathy, we irradiated the whole abdomen of the mice with 13.5 Gy of X-ray and administered ginsenoside Rb1 intraperitoneally at 20 mg/kg/day for 6 days (Fig. 1A). The bodyweight of the IR group, which gradually decreased from the day of radiation exposure, was significantly recovered in the Rb1 group (Fig. 1B). The Rb1 group exhibited reduced histological damage on the small intestine, including decreased villus height, and crypt loss, compared to the IR group (Fig. 1C and D). Similarly, the histological scoring parameters reflecting epithelial and crypt injury, vascular dilation, and inflammatory changes, were markedly reduced in the Rb1 group compared with those in the IR group (Fig. 1D). The bacterial colony counts in MLN tissues, an indicator of bacterial translocation to other organs, were increased in the IR group compared with those in the NC group, and were markedly reduced in the Rb1 group compared with those in the IR group (Fig. 1E). To assess the effect of Rb1 on intestinal permeability, an FITC–dextran assay was performed. The elevated FITC concentration in the IR group was significantly reduced following Rb1 administration (Fig. 1F). The mRNA levels of *Villin*, *Cldn3,* and occludin (*Ocln*), which contribute to the epithelial barrier, were markedly increased in the small intestines of the Rb1 group compared with those in the small intestine of the IR group (Fig. 1G). Histological evaluation of the epithelial integrity (villin, a marker of enterocytes) and the intestinal barrier (CLDN3, a tight junction molecule) was improved by the Rb1 administration (Fig. 1H). These results suggest that Rb1 mitigates acute radiation-induced intestinal injury with improved epithelial barrier function.

### 3.2 Ginsenoside Rb1 regulated inflammatory response and increased Treg cell population in radiation-induced intestinal injury

**Fig. 2:**
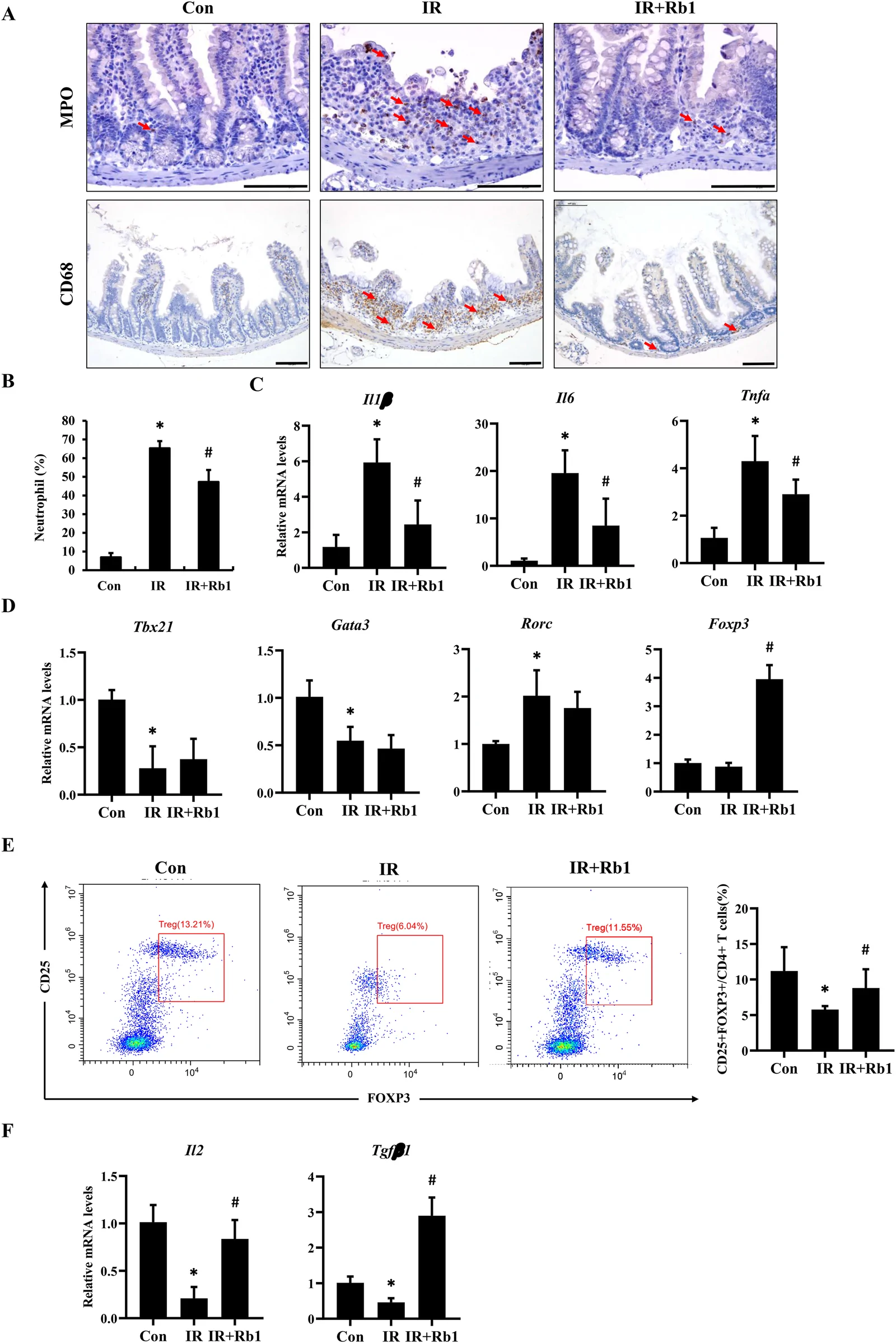
Ginsenoside Rb1 regulated inflammatory response and increased Treg cell population in radiation-induced intestinal injury. (A) Representative IHC staining of myeloperoxidase (MPO) and CD68 in the small intestine. Red arrows indicate positive cells. Scale bar = 100 μm. (B) Percentage of neutrophils in the plasma. (C) mRNA levels of *Il1β, Il6,* and *Tnfα* and (D) lineage-defining transcription factors (*Tbx21, Gata3, Rorc,* and *Foxp3*). (E) Percentages of CD25^+^FOXP3^+^Treg determined in the CD4^+^ T cell population. CD25^+^FOXP3^+^/CD4^+^ T cells ratio in the lamina propria was also calculated. (F) mRNA levels of Treg-associated cytokines (*Il2* and *Tgfβ1*). Values are shown as means ± standard deviations (*n* = 6). Statistical significance was determined using one-way ANOVA followed by Tukey's post hoc test. *p < 0.05 vs. Con group; ^#^*p* < 0.05 vs. IR group.

We investigated the effects of Rb1 on innate and adaptive immune responses in radiation-induced enteritis. The IR group showed increased infiltration of neutrophils (MPO) and macrophages (CD68) in the intestine compared with that in the NC group (Fig. 2A). However, Rb1 treatment markedly reduced the number of MPO- and CD68-positive cells in the irradiated small intestines (Fig. 2A). Circulating neutrophils serve as systemic indicators of inflammation, and in our study, radiation exposure markedly increased blood neutrophil levels (Fig. 2B). However, this elevation was significantly attenuated in the Rb1-treated group (Fig. 2B). The mRNA levels of inflammatory cytokines, such as *Il1β, Il6*, and *Tnfα* were significantly increased in the IR group compared with those in the NC group, and statistically decreased in the Rb1 group compared with those in the IR group (Fig. 2C). In the adaptive immune response, T cells are central mediators; among them, CD4^+^ T cells coordinate immune responses and promote tissue repair by secreting various cytokines [22]. The major CD4^+^ T cell subsets include Th1 (*Tbx21*), Th2 (*Gata3*), Th17 (*Rorc*), and Treg (*Foxp3*) cells. Radiation exposure altered the expression of multiple CD4^+^ T cell lineage-associated transcription factors, including decreased *Tbx21* and *Gata3* expression and increased *Rorc* expression. Among these transcription factors, Rb1 selectively increased *Foxp3* expression without significantly affecting *Tbx21, Gata3*, or *Rorc* expression (Fig. 2D). Moreover, the proportion of FOXP3^+^CD25^+^ Treg cells in the lamina propria of the small intestine was reduced in the IR group, whereas this reduction was significantly reversed by Rb1 administration (Fig. 2E). The analysis of mRNA expression revealed that *Il2 and Tgfβ1*, key factors associated with Treg cell differentiation and maintenance, were significantly upregulated in the Rb1 group compared with that in the IR group (Fig. 2F). These results suggest that Rb1 treatment reduced the inflammatory response and increased the Treg cell population in radiation-induced intestinal injury.

### 3.3 Ginsenoside Rb1 treatment enhanced epithelial regeneration with the Wnt/β-catenin signaling pathway in radiation-induced intestinal injury

**Fig. 3:**
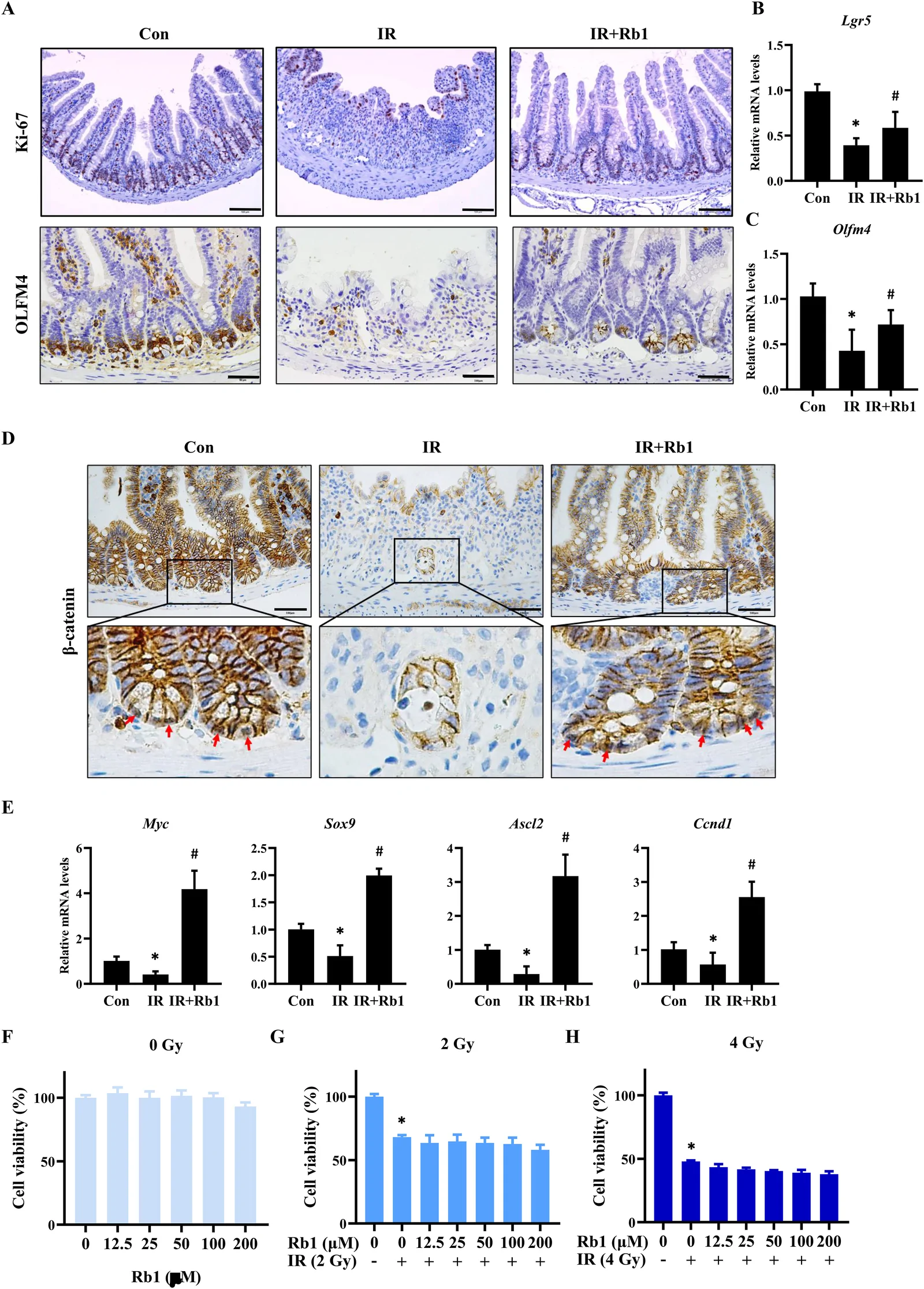
Ginsenoside Rb1 treatment enhanced epithelial regeneration with the Wnt/β-catenin signaling pathway in radiation-induced intestinal injury. (A) Representative IHC staining of Ki-67 and OLFM4 in the small intestine. Scale bar = 50 μm. mRNA levels of (B) *Lgr5* and (C) *Olfm4*. (D) Representative IHC staining of β-catenin in the small intestine. Scale bar = 50 μm. (E) mRNA levels of *Myc, Sox9, Ascl2,* and *Ccnd1*. (F-H) Cell viability of IEC-6 cells treated with Rb1 after irradiation with 0, 2, and 4 Gy. Values are shown as means ± standard deviations (*n* = 6). Statistical significance was determined using one-way ANOVA followed by Tukey's post hoc test. *p < 0.05 vs. Con group; ^#^*p* < 0.05 vs. IR group.

To determine whether Rb1 administration improved epithelial damage, the protein expression levels of proliferation-related factors and pathways in the small intestine were assessed. The proliferative epithelial cells (Ki-67) and ISC (Olfm4) were markedly upregulated in the small intestine of the Rb1 group compared to the that of the IR group (Fig. 3A). Consistently, Rb1 significantly restored the mRNA levels of the ISC markers *Lgr5* and *Olfm4* relative to those in the IR group (Fig. 3B and C). The epithelial expression and translocation to the nucleus of β-catenin, a key factor in the Wnt/β-catenin pathway, was markedly downregulated in the IR group and restored by the administration of Rb1 (Fig. 3D). Additionally, the mRNA expression levels of *Myc, Sox9, Ascl2*, and *Ccnd1*, which are involved in the Wnt/β-catenin signaling pathway were significantly upregulated in the small intestine of the Rb1 group compared with those in the IR group (Fig. 3E). We subsequently evaluated the proliferative response of intestinal epithelial cells to Rb1. Cell viability assays confirmed that Rb1 was not cytotoxic at concentrations up to 200 μM and did not exhibit proliferative activity in IEC-6 cells irradiated with 2 or 4 Gy (Fig. 3F-H). These results suggest that Rb1 promotes epithelial regeneration following radiation-induced intestinal injury through activation of the Wnt/β-catenin signaling pathway.

### 3.4 IL-10 treatment improved epithelial cell proliferation via the Wnt/β-catenin signaling pathway in irradiated IEC-6 cells

**Fig. 4:**
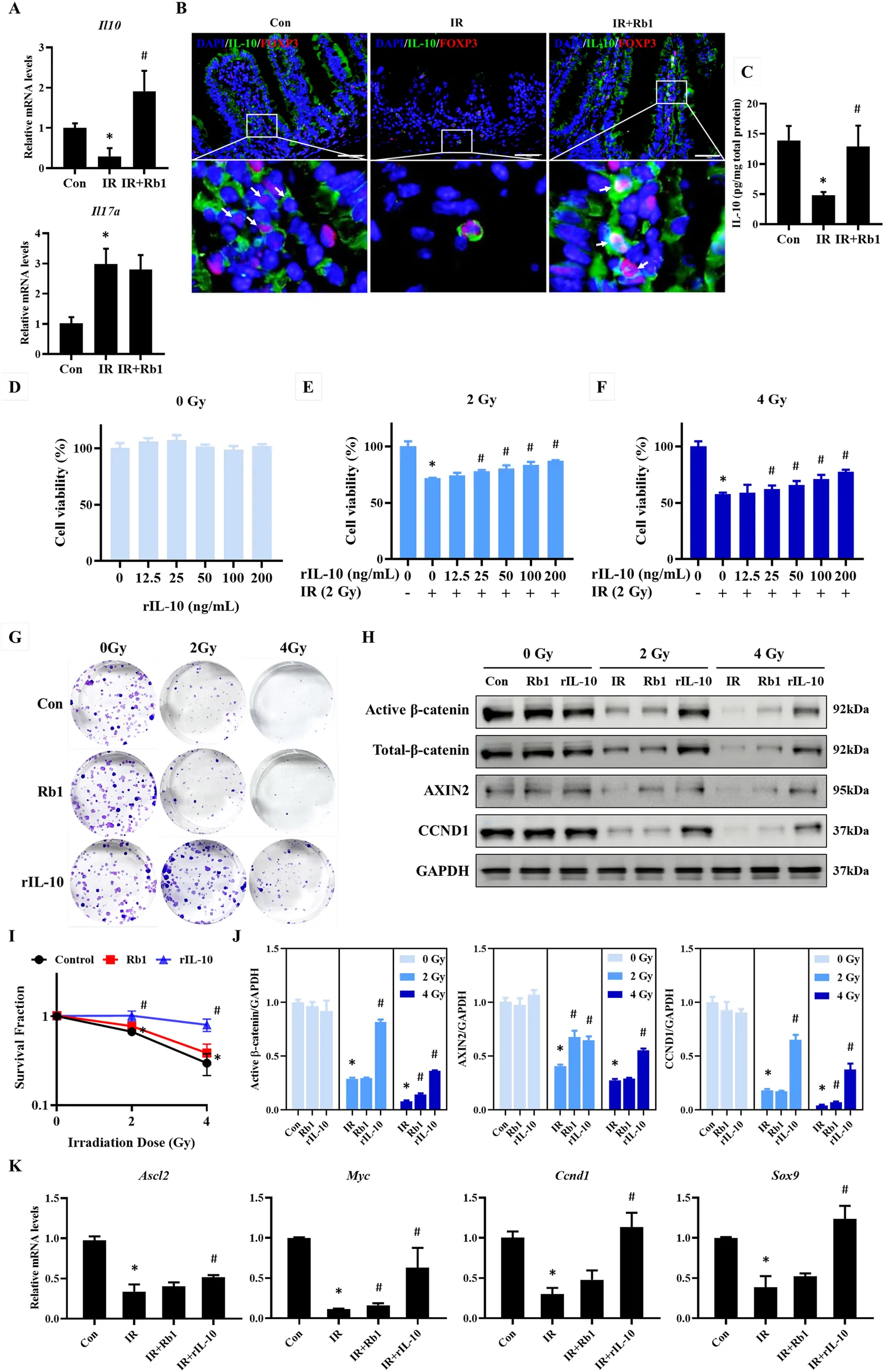
IL-10 treatment improved epithelial cell proliferation via the Wnt/β-catenin signaling pathway in irradiated IEC-6 cells. (A) mRNA level of *Il10* and *Fgf2*. (B) Representative immunofluorescence images of IL-10 (green), FOXP3 (red), and DNA (blue). White arrows indicate IL-10/FOXP3 double-positive cells. Scale bar = 50 μm. (C) IL-10 levels in ileal tissue homogenates were quantified by ELISA. (D-F) Cell viability of IEC-6 cells treated with rIL-10 after irradiation with 0, 2, and 4 Gy. (G) Representative colony formation images and (I) the survival fraction of irradiated IEC-6 cells treated with Rb1 or rIL-10. (H) Representative immunoblot images and (J) densitometric analysis of active β-catenin, total β-catenin, AXIN2, CCND1, and GAPDH. (K) mRNA levels of *Ascl2, Myc, Ccnd1*, and *Sox9*. Values are shown as means ± standard deviations (*n* = 3). Statistical significance was determined using one-way ANOVA followed by Tukey's post hoc test. *p < 0.05 vs. Con group; ^#^*p* < 0.05 vs. IR group.

As Rb1 did not directly promote intestinal epithelial proliferation, we focused on the increase in Treg cells observed following Rb1 treatment. Treg cells not only modulate inflammation but also promote epithelial regeneration through the secretion of soluble mediators [23]. Therefore, we evaluated the mRNA expression levels of *Fgf2* and *Il10*, which are Treg-associated soluble factors implicated in epithelial cell proliferation [23]. Rb1 did not significantly increase the mRNA levels of Fgf2, whereas Il10 expression was markedly increased in the irradiated intestine following Rb1 treatment (Fig. 4A). Immunofluorescence analysis revealed increased IL-10 signals in intestinal tissues, frequently observed in association with FOXP3^+^ cells (Fig. 4B). To further confirm IL-10 upregulation at the protein level, we performed ELISA using ileal tissue lysates. Consistent with the mRNA and immunofluorescence results, IL-10 protein levels were significantly increased in the IR + Rb1 group compared with the IR group (Fig. 4C). To determine whether IL-10 directly affected radiation-induced epithelial injury, we performed an in vitro assay using irradiated intestinal epithelial cells. No cytotoxicity was observed with rIL-10 at concentrations up to 200 ng/mL, and rIL-10 alleviated radiation-induced cytotoxicity (2 or 4 Gy) in a concentration-dependent manner (Fig. 4D−4F). Based on the cytotoxicity results, subsequent in vitro experiments were conducted using 100 μM Rb1 and 100 ng/mL rIL-10. Based on the colony formation results, rIL-10 significantly improved the survival and clonogenicity of IEC-6 cells irradiated at 2 and 4 Gy compared to the control group, whereas the effects of Rb1 were negligible (Fig. 4G,I). Western blot analysis showed that rIL-10 treatment significantly increased the expression of Wnt/β-catenin signaling factors, including active β-catenin, AXIN2, and CCND1, which were reduced by irradiation (Fig. 4H,J). Consistent with these findings, rIL-10 also markedly restored the mRNA levels of canonical Wnt/β-catenin target genes, such as *Ascl2, Myc, Ccnd1*, and *Sox9* (Fig. 4K). Thus, IL-10, whose expression was upregulated in Rb1-treated Treg cells, could directly contribute to epithelial proliferation following irradiation and activates the Wnt/β-catenin signaling pathway.

### 3.5 Ginsenoside Rb1 alleviated radiation-induced intestinal injury through Treg-associated IL-10 signaling

**Fig. 5:**
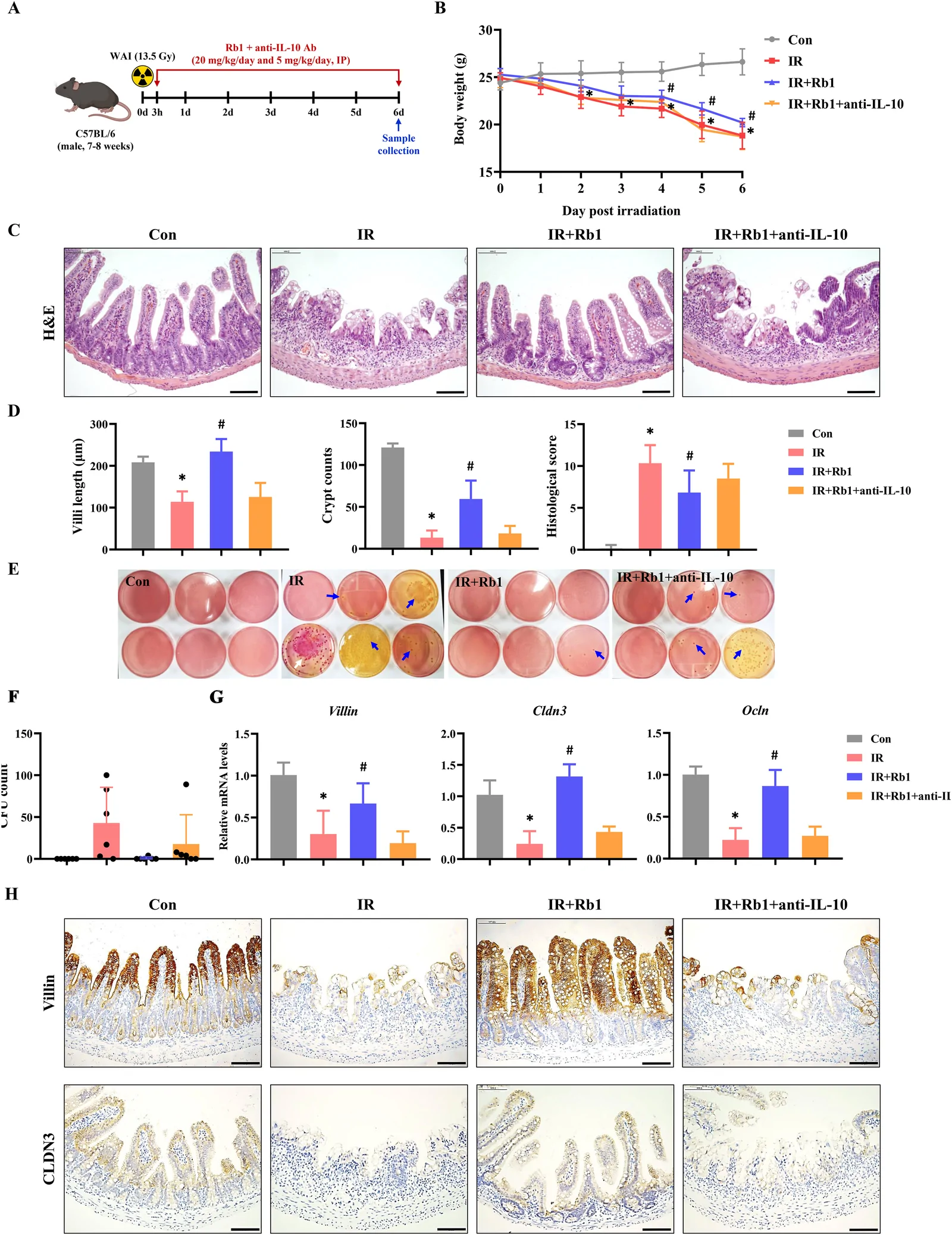
Ginsenoside Rb1 alleviated radiation-induced intestinal injury through IL-10–dependent signaling.(A) Experimental scheme: mice received abdominal irradiation (13.5 Gy) followed by Rb1 (20 mg/kg) and anti-IL-10 (5 mg/kg) for 6 days. (B) Body weight changes of control (Con), irradiated (IR), Rb1-treated irradiated (IR + Rb1), and Rb1 and anti-IL-10-treated irradiated mice. (C) H&E staining of the small intestine. Scale bar = 100 μm. (D) Villi length, crypt counts, and histological score of the small intestines. (E) Representative images of bacterial colonies cultured from mesenteric lymph nodes and (F) quantification of colony-forming units (CFUs) per mesenteric lymph nodes sample. (G) mRNA levels of *Villin, Cldn3,* and *Ocln* in the small intestine. (H) Representative IHC staining of villin and CLDN3. Scale bar = 100 μm. Values are shown as means ± standard deviations (*n* = 6). Statistical significance was determined using one-way ANOVA followed by Tukey's post hoc test. *p < 0.05 vs. Con group; ^#^*p* < 0.05 vs. IR group.

To determine whether IL-10 mediates the therapeutic role of Rb1, we administered an IL-10 neutralizing antibody intraperitoneally in combination with Rb1 to irradiated mice for 6 days (Fig. 5A). Rb1 treatment significantly restored the irradiation-induced body weight loss. However, this effect was abolished by IL-10 neutralization (Fig. 5B). Histological analysis further demonstrated that the IL-10 blockade negated the therapeutic effects of Rb1, as indicated by persistent epithelial and crypt injury, villus shortening, and inflammatory changes (Fig. 5C and D). These observations were confirmed by quantitative measurements of villus length and crypt number (Fig. 5D). Consistently, bacterial translocation assays revealed that the Rb1-mediated barrier recovery was reversed on IL-10 neutralization (Fig. 5E and F). Moreover, the Rb1-induced improvements in epithelial barrier integrity and upregulation of tight junction factors were abrogated by IL-10 neutralization, at both the mRNA and protein levels (Fig. 5G and H). These results demonstrate that IL-10 signaling associated with Treg expansion is essential for the therapeutic effects of Rb1 against radiation-induced intestinal injury, including epithelial integrity and barrier function.

### 3.6 Ginsenoside Rb1 promotes intestinal epithelial regeneration through Treg-associated IL-10 signaling

**Fig. 6:**
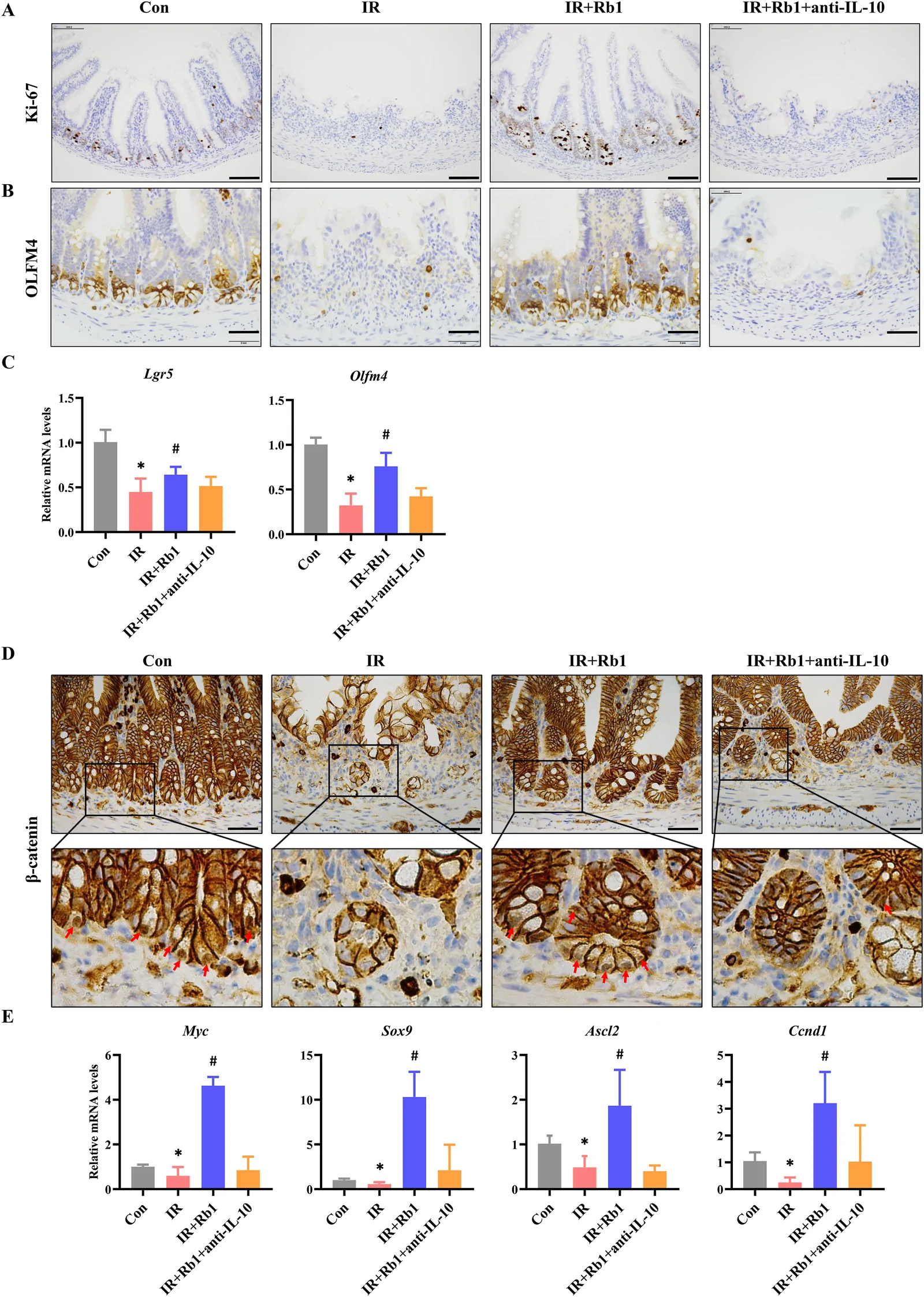
Ginsenoside Rb1 promotes intestinal epithelial regeneration through IL-10–mediated signaling. Representative IHC staining of (A) Ki-67 and (B) OLFM4 in the small intestine. (C) mRNA levels of *Lgr5* and *Olfm4*. (D) Representative IHC staining of β-catenin in the small intestine. Red arrow indicates the nuclear translocated β-catenin. Scale bar = 100 μm. (E) mRNA levels of *Myc, Sox9, Ascl2*, and *Ccnd1*. Values are shown as means ± standard deviations (n = 6). Statistical significance was determined using one-way ANOVA followed by Tukey's post hoc test. *p < 0.05 vs. Con group; #p < 0.05 vs. IR group.

In terms of epithelial regeneration, Rb1 treatment markedly enhanced several regenerative markers in the irradiated intestines. Ki-67 expression, which reflects the proliferative activity of crypt epithelial cells, was significantly increased by Rb1 treatment, indicating that crypt cell proliferation was effectively restored (Fig. 6A). However, this proliferative effect was markedly reduced when IL-10 was neutralized (Fig. 6A). *Olfm4* and *Lgr5*, which were specific markers of ISCs, were upregulated by Rb1, indicating their role in preserving and expanding the stem cell compartment, whereas this increase was markedly reduced in the presence of the IL-10 antibody (Fig. 6B and C). Rb1 markedly increased the expression of β-catenin expression and translocation to the nucleus in the small intestine, indicating activation of Wnt/β-catenin signaling, but this effect was almost abolished by IL-10 neutralization (Fig. 6D). In addition, the mRNA expression of canonical Wnt/β-catenin target genes, including *Myc, Sox9, Ascl2,* and *Ccnd1*, was significantly elevated by Rb1 treatment (Fig. 6E). However, these transcriptional increases were statistically suppressed upon IL-10 neutralization (Fig. 6E). Together, these results indicate that the ability of Rb1 to promote epithelial proliferation, maintain intestinal stemness, and activate Wnt/β-catenin–driven regenerative signaling is critically dependent on IL-10.

## 4 Discussion

Advances in radiation technology have reinforced their central role in cancer therapy and diagnostic imaging. However, effective radioprotective or therapeutic agents are still urgently needed to mitigate normal tissue injury caused by radiation exposure [24,25]. Among tissues affected by radiation, epithelial cells are particularly susceptible because their continuous proliferation makes them highly sensitive to genotoxic stress [4]. Radiation-induced intestinal injury was accompanied by pathological features such as body weight loss, crypt destruction, villus shortening, bacterial translocation due to loss of tight junction integrity, and inflammatory cell infiltration [26]. We demonstrated that ginsenoside Rb1 markedly promotes the repair of radiation-induced intestinal epithelial damage. Mechanistically, Rb1 increased the population of Treg cells and promoted the secretion of the anti-inflammatory cytokine IL-10, which contributed to the activation of the Wnt/β-catenin signaling pathway in the irradiated epithelial cells. The activation of Wnt/β-catenin pathway by IL-10 enhanced the proliferative capacity of intestinal epithelial cells, thereby reinforcing epithelial barrier function. These results indicate that Rb1 may serve as a promising therapeutic candidate for radiation-induced enteropathy.

Among the major bioactive ginsenosides in red ginseng, Rg1, Rb1, and Rg3 have been extensively studied for their diverse pharmacological activities [14]. In our screening analysis, only Rb1 showed a significant therapeutic effect against radiation-induced intestinal injury. Given that previous studies have demonstrated the anti-inflammatory and intestinal barrier-protective properties of Rb1 in other models of intestinal damage, our findings suggest that Rb1 may be a particularly relevant ginsenoside for mitigating radiation-induced intestinal injury. Ginsenoside Rb1 is the main bioactive constituent of *Panax ginseng*, predominantly distributed in its roots, stems, and flower buds [13]. Rb1 exerts diverse pharmacological effects, such as anti-inflammatory, antioxidant, immunoregulatory, anti-obesity, anti-hyperglycemic, and neuroprotective effects, by modulating various cellular signaling pathways [14,17,27]. Notably, recent studies have demonstrated that Rb1 attenuates colitis in DSS-induced ulcerative colitis mouse models by modulating HRD1-mediated ER homeostasis in LPS-treated epithelial cells and NRF2-dependant barrier reinforce [13,17]. Also, Zhou et al. [18] have reported that Rb1 ameliorates intestinal barrier dysfunction and upregulates the production of anti-inflammatory cytokines. Furthermore, evidence from murine models and human specimens from patients with asthma indicates that Rb1 facilitates Treg cell population and IL-10 production from Treg cells [16]. These findings support the therapeutic potential of Rb1 in recovering intestinal epithelial integrity and modulating immune responses in radiation-induced intestinal injury. In this study, Rb1 enhanced epithelial regeneration, including barrier function and stem cell conservation and regulated inflammatory responses with increased Treg cell population, possibly via increased IL-2 production by CD4^+^ T cells and TGF-β1 secretion by epithelial cells. Furthermore, we identified IL-10 as a key target mediator of epithelial repair and immune regulatory effects.

IL-10, which is primarily produced by Treg with anti-inflammatory functions, is markedly reduced in intestinal tissues following abdominal irradiation [28,29]. As a critical immune regulator, IL-10 functions as a potent immunoregulatory cytokine that restrains the production of pro-inflammatory mediators, including IL-1, IL-6, and TNF-α, and its deficiency has been associated with severe enteritis in children [30]. Also, Li et al. [31] have reported that the regulation of IL-10 has therapeutic potential in human inflammatory bowel disease. Xie et al. [29] have reported that microbiota-mediated metabolites increase epithelial IL-10 synthesis and accelerate intestinal epithelial repair and regeneration through stimulation of Wnt/β-catenin signaling. Our findings demonstrated that IL-10 contributed to the repair of radiation-induced epithelial damage. Although Rb1 itself did not directly enhance the proliferation of IEC-6 cells, IL-10 activated the Wnt/β-catenin signaling cascade, ultimately promoting IEC-6 cell proliferation and clonogenic survival. While Rb1 partially affected the expression of several Wnt/β-catenin target genes in IEC-6 cells, these transcriptional changes were not accompanied by measurable functional effects on epithelial proliferation or clonogenic survival, suggesting limited direct biological activity in epithelial cells. Importantly, neutralization of IL-10 almost completely abrogated the therapeutic effect of Rb1, highlighting IL-10 as an indispensable mediator of Rb1-driven intestinal protection.

Maintenance of the epithelium is dependent on the coordinated activity of signaling pathways, particularly the Wnt, Notch, and Hippo pathways [32]. Notably, the Wnt/β-catenin signaling pathway engages with the effectors, including *Myc, Ccnd1, Axin2, Ascl2*, and *Sox9*, thereby exerting significant control over epithelial cell proliferation and differentiation, which are fundamental to preserving intestinal barrier integrity [33,34]. Accumulating evidence indicates that loss of β-catenin signaling has been linked to epithelial injury in various intestinal disorders, whereas restoration of its nuclear activity promotes regeneration of impaired enterocytes. [35,36]. Xie et al. [29] have reported that the IL-10/Wnt/β-catenin axis facilitates crypt stem cell proliferation and regeneration in radiation-induced intestinal injury. We observed that β-catenin was stabilized and translocated into the nucleus in response to Rb1-mediated IL-10, ultimately promoting the restoration of intestinal epithelial proliferation.

This study has several limitations. Only a single dose of Rb1 was evaluated in vivo, and dose–response relationships were not systematically examined. Our analysis focused primarily on the acute phase of radiation-induced intestinal injury, and therefore the long-term therapeutic effects of Rb1, including survival outcomes and chronic intestinal pathology, were not assessed. In addition, although Tgfβ1 expression may support Treg-associated immune regulation during acute intestinal injury, long-term studies evaluating potential fibrotic outcomes following Rb1 treatment would be valuable. Although our data indicate that IL-10 plays a critical role in the therapeutic effects of Rb1, the precise cellular mechanism by which IL-10 promotes intestinal stem cell activity and epithelial regeneration remains incompletely defined, and the potential contribution of other IL-10–producing cell populations cannot be fully excluded. Definitive identification of the cellular source of IL-10 would require additional approaches such as intracellular cytokine staining or lineage-tracing analyses. In addition, direct mechanistic validation of IL-10-mediated Wnt/β-catenin activation using Wnt inhibitors or IL-10R blockade in epithelial cells was not performed and remains an important topic for future investigation. Although FOXP3^+^CD25^+^ Treg populations were increased following Rb1 treatment, functional characterization of suppressive Treg activity was not performed. Further analyses using suppression assays or additional Treg-associated markers would strengthen the characterization of the Treg phenotype. The in vitro experiments did not include a conventional positive control, as there is currently no widely established pharmacological agent routinely used as a positive control for epithelial regeneration in radiation-induced intestinal injury models. These experiments were primarily designed to evaluate the mechanistic role of IL-10 signaling rather than to compare therapeutic efficacy. In addition, the in vivo and in vitro experiments were performed using different rodent species, although IL-10 receptor signaling is highly conserved across rodents. Finally, Rb1 was administered intraperitoneally to ensure consistent systemic delivery. However, this route may limit direct translational relevance. Future studies evaluating oral administration, clinically relevant ginseng-derived formulations, and long-term outcomes will be necessary to further assess the therapeutic potential and mechanism of Rb1.

## 5 Conclusion

Rb1 alleviated radiation-induced intestinal injury by promoting epithelial regeneration associated with activation of the Wnt/β-catenin pathway and increased IL-10 signaling. Taken together, our findings suggest that Rb1 enhances intestinal repair in the context of Treg expansion and IL-10–mediated immune–epithelial interactions, highlighting its potential as a therapeutic candidate for radiation-induced enteropathy.

## Declaration of competing interest

The authors declare no conflict of interest.
